# (*E*)-3-(8-Benz­yloxy-2,3-dihydro-1,4-benzodioxin-6-yl)-1-[2-hy­droxy-4,6-bis­(meth­oxy­meth­oxy)phen­yl]prop-2-en-1-one

**DOI:** 10.1107/S160053681100924X

**Published:** 2011-03-19

**Authors:** Yu Zhang, Yi-Nan Zhang, Ming-Ming Liu, Kum-Chol Ryu, De-Yong Ye

**Affiliations:** a Department of Medicinal Chemistry, School of Pharmacy, Fudan University, Shanghai 201203, People’s Republic of China; b Institute of Pharmacy, Ham Hung Pharmaceutical University, Ham Hung, Democratic People’s Republic of Korea

## Abstract

In the title mol­ecule, C_28_H_28_O_9_, the phenol and the benzene rings adjacent to the α,β-unsaturated ketone unit are inclined at 9.15 (13)° to each other. The terminal phenyl ring is oriented with respect to the phenol ring at a dihedral angle of 85.88 (13)°. In the crystal, the methyl­ene C atoms of the dihydro­dioxine ring are disordered over two sites with an occupancy ratio of 0.463 (18):0.537 (18), and both disordered components of the dihydro­dioxine ring adopt twisted-chair conformations. An intra­molecular O—H⋯O hydrogen bond and weak inter­molecular C—H⋯O hydrogen bonds are present in the crystal structure.

## Related literature

For background to 1,3-diaryl-2-propen-1-one, see: Carlo *et al.* (1999 ▶); Dimmock *et al.* (1999 ▶); Go *et al.* (2005 ▶); Nowakowska (2007 ▶); Yarishkin *et al.* (2008 ▶). For related structures, see: Özbey *et al.* (1997 ▶); Gao & Ng (2006 ▶); Loghmani-Khouzani *et al.* (2009 ▶); Rizvi *et al.* (2010 ▶). For the synthesis, see: Lin *et al.* (2007 ▶).
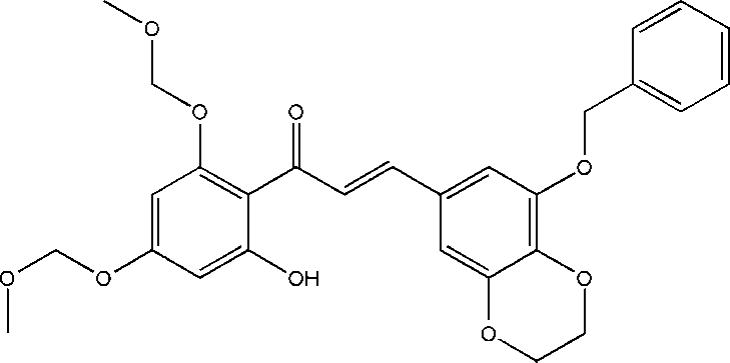


## Experimental

### 

#### Crystal data

C_28_H_28_O_9_
*M* *_r_* = 508.50
Triclinic, 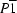

*a* = 8.149 (4) Å
*b* = 11.744 (5) Å
*c* = 14.439 (7) Å
α = 72.752 (5)°
β = 84.269 (5)°
γ = 70.601 (5)°
*V* = 1244.7 (10) Å^3^
*Z* = 2
Mo *K*α radiation
μ = 0.10 mm^−1^
*T* = 294 K
0.35 × 0.25 × 0.18 mm

#### Data collection

Bruker CCD 1000 area-detector diffractometer
Absorption correction: multi-scan (*SADABS*; Sheldrick, 1996 ▶) *T*_min_ = 0.965, *T*_max_ = 0.982
5687 measured reflections
4774 independent reflections
3239 reflections with *I* > 2σ(*I*)
*R*_int_ = 0.026

#### Refinement

*R*[*F* ^2^ > 2σ(*F* ^2^)] = 0.061
*wR*(*F* ^2^) = 0.195
*S* = 1.02
4774 reflections
353 parameters
H-atom parameters constrained
Δρ_max_ = 0.60 e Å^−3^
Δρ_min_ = −0.21 e Å^−3^

### 

Data collection: *SMART* (Bruker, 2001 ▶); cell refinement: *SAINT* (Bruker, 2001 ▶); data reduction: *SAINT*; program(s) used to solve structure: *SHELXTL* (Sheldrick, 2008 ▶); program(s) used to refine structure: *SHELXTL*; molecular graphics: *SHELXTL*; software used to prepare material for publication: *SHELXTL*.

## Supplementary Material

Crystal structure: contains datablocks I, global. DOI: 10.1107/S160053681100924X/xu5171sup1.cif

Structure factors: contains datablocks I. DOI: 10.1107/S160053681100924X/xu5171Isup2.hkl

Additional supplementary materials:  crystallographic information; 3D view; checkCIF report

## Figures and Tables

**Table 1:** Hydrogen-bond geometry (Å, °)

*D*—H⋯*A*	*D*—H	H⋯*A*	*D*⋯*A*	*D*—H⋯*A*
O9—H9*C*⋯O4	0.82	1.71	2.448 (2)	149
C6—H6*A*⋯O9^i^	0.93	2.57	3.426 (3)	154
C21—H21*A*⋯O8^ii^	0.93	2.53	3.453 (3)	169

Symmetry codes: (i) 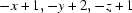; (ii) 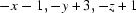.

**Fig. 1.**
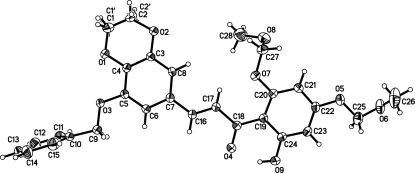
The molecular structure of title compound. The displacement ellipsoids are drawn at the 50% probability level. H atoms are presented as small spheres of arbitrary radius.

## supplementary crystallographic information

### Comment

1,3-Diaryl-2-propen-1-ones, commonly known as chalcones are a kind of aromatic ketones that form the central core for a variety of important biological compounds, showing anti-bacterial, anti-fungal, anti-malarial, anti-viral, anti-inflammatory, anti-oxidant and anti-tumor properties, which have been reviewed by Carlo *et al.* (1999), Dimmock *et al.* (1999), Go *et al.* (2005) and Nowakowska (2007). Some even demonstrated the ability to block voltage-dependent potassium channels (Yarishkin *et al.*, 2008). Chalcones can also be converted into flavonoids in several classical synthetic steps, consolidating its significance in synthetic chemistry.

The title compound, *i.e.* (*E*)-3-(8-(benzyloxy)-2,3-dihydrobenzo[*b*][1,4]dioxin-6-yl) -1-(2-hydroxy-4,6-bis(methoxymethyl)phenyl)prop-2-en-1-one, is first prepared aiming to find out potential anti-virus candidates. It is obtained from a dihydrobenzo[*b*][1,4]dioxine aldehyde derivative and an acetophenone derivative by a classical aldol condensation in the presence of potassium hydroxide as a catalyst (Lin *et al.*, 2007). We now report here the synthesis and crystal structure, as part of our investigations in revealing the relationship between the structure and the anti-virus activity. A series of chalcones related to the title compound is also under investigation for their biological activities in our laboratory.

The title molecule is presented in Fig. 1. The least-square planes of the benzene and phenol rings defined by atoms C3—C8 and C19—C24, respectively, are inclined at 9.15 (13)° with respect to each other. The dihedral angle between the benzyloxy group and the benzene plane is 85.90 (7)°. Both the major and minor conformers of the disordered dioxane ring adopt twist-chair conformations [φ = 193.86 (8)°, θ = 77.63 (16)° (for ring C1—C2—O2—C3—C4—O1), and φ = 184.02 (10)°, θ = 95.1 (2)° (for ring C1'-C2'-O2—C3—C4—O1)], having total puckering amplitudes, Q_T_, of 0.2538 (8) Å and 0.1675 (10) Å, respectively. The crystal structure is stabilized by a strong intramolecular O—H···O hydrogen bond, and further consolidated by the weak intermolecular hydrogen-bonding interactions of the type C—H···O, and Van der Waals forces (Table 1).

### Experimental

1-[2-Hydroxy-4,6-bis-(methoxymethyl)-phenyl]-ethanone (1.8 g, 7.0 mmol) and 8-(benzyloxy)-2,3-dihydro-benzo[*b*][1,4]dioxine-6-carbaldehyde (1.9 g, 7.0 mmol) were dissolved in 95% EtOH, KOH/H_2_O solution (3*M*). The reaction mixture was stirred at room temperature overnight, evaporated under reduced pressure, and further extracted by ethyl acetate. The extract layer was chromatographed on silica gel to afford the title compound (3.2 g, yield 90%) as an orange solid. Single crystals suitable for X-ray analysis were obtained by slow evaporation of an ethyl acetate and petroleum ether solution.

### Refinement

The methylene C atoms of the dihydrodioxine ring are disordered over two sites (C1/C1' and C2/C2') with refined occupancies of 0.463 (18):0.537 (18). The corresponding bond distances involving the disordered atoms were restrained to be equal, and also the same U^ij^ parameters were used for atoms C1 and C1', and C2 and C2'. All H atoms were positioned geometrically with C—H = 0.93–0.97 Å, O—H = 0.82 Å, and refined as riding with *U*_iso_(H) = 1.2–1.5*U*_eq_(C,O).

### Crystal data

**Table:**

C_28_H_28_O_9_	*Z* = 2
*M**_r_* = 508.50	*F*(000) = 536
Triclinic, *P*1	*D*_x_ = 1.357 Mg m^−^^3^
Hall symbol: -P 1	Mo *K*α radiation, λ = 0.71073 Å
*a* = 8.149 (4) Å	Cell parameters from 847 reflections
*b* = 11.744 (5) Å	θ = 2.7–27.1°
*c* = 14.439 (7) Å	µ = 0.10 mm^−^^1^
α = 72.752 (5)°	*T* = 294 K
β = 84.269 (5)°	Block, orange
γ = 70.601 (5)°	0.35 × 0.25 × 0.18 mm
*V* = 1244.7 (10) Å^3^	

### Data collection

**Table:**

Bruker CCD 1000 area-detector diffractometer	4774 independent reflections
Radiation source: sealed tube	3239 reflections with *I* > 2σ(*I*)
graphite	*R*_int_ = 0.026
φ and ω scans	θ_max_ = 26.0°, θ_min_ = 1.5°
Absorption correction: multi-scan (*SADABS*; Sheldrick, 1996)	*h* = −9→10
*T*_min_ = 0.965, *T*_max_ = 0.982	*k* = −14→12
5687 measured reflections	*l* = −17→17

### Refinement

**Table:**

Refinement on *F*^2^	Primary atom site location: structure-invariant direct methods
Least-squares matrix: full	Secondary atom site location: difference Fourier map
*R*[*F*^2^ > 2σ(*F*^2^)] = 0.061	Hydrogen site location: inferred from neighbouring sites
*wR*(*F*^2^) = 0.195	H-atom parameters constrained
*S* = 1.02	*w* = 1/[σ^2^(*F*_o_^2^) + (0.126*P*)^2^] where *P* = (*F*_o_^2^ + 2*F*_c_^2^)/3
4774 reflections	(Δ/σ)_max_ < 0.001
353 parameters	Δρ_max_ = 0.60 e Å^−^^3^
0 restraints	Δρ_min_ = −0.21 e Å^−^^3^

### Special details

**Table:**

Geometry. All e.s.d.'s (except the e.s.d. in the dihedral angle between two l.s. planes) are estimated using the full covariance matrix. The cell e.s.d.'s are taken into account individually in the estimation of e.s.d.'s in distances, angles and torsion angles; correlations between e.s.d.'s in cell parameters are only used when they are defined by crystal symmetry. An approximate (isotropic) treatment of cell e.s.d.'s is used for estimating e.s.d.'s involving l.s. planes.
Refinement. Refinement of *F*^2^ against ALL reflections. The weighted *R*-factor *wR* and goodness of fit *S* are based on *F*^2^, conventional *R*-factors *R* are based on *F*, with *F* set to zero for negative *F*^2^. The threshold expression of *F*^2^ > σ(*F*^2^) is used only for calculating *R*-factors(gt) *etc*. and is not relevant to the choice of reflections for refinement. *R*-factors based on *F*^2^ are statistically about twice as large as those based on *F*, and *R*- factors based on ALL data will be even larger.

### Fractional atomic coordinates and isotropic or equivalent isotropic displacement parameters (Å^2^)

**Table:**

	*x*	*y*	*z*	*U*_iso_*/*U*_eq_	Occ. (<1)
O1	−0.02313 (19)	0.61404 (14)	0.91280 (12)	0.0582 (5)	
O2	−0.2521 (2)	0.86192 (14)	0.83479 (12)	0.0597 (5)	
O3	0.29103 (19)	0.54252 (13)	0.84269 (11)	0.0558 (5)	
O4	0.3240 (2)	1.09630 (15)	0.44508 (13)	0.0642 (5)	
O5	−0.1587 (2)	1.65623 (15)	0.28563 (12)	0.0647 (5)	
O6	−0.2044 (3)	1.83191 (15)	0.15912 (13)	0.0736 (5)	
O7	−0.1328 (2)	1.30029 (15)	0.55058 (12)	0.0659 (5)	
O8	−0.4304 (3)	1.35542 (18)	0.53966 (16)	0.0848 (6)	
O9	0.3228 (2)	1.28722 (16)	0.31656 (13)	0.0668 (5)	
H9C	0.3572	1.2137	0.3494	0.100*	
C1	−0.172 (2)	0.6699 (16)	0.9639 (10)	0.086 (4)	0.463 (18)
H1A	−0.2494	0.6200	0.9719	0.103*	0.463 (18)
H1B	−0.1333	0.6586	1.0283	0.103*	0.463 (18)
C2	−0.2644 (15)	0.7837 (9)	0.9331 (7)	0.057 (2)	0.463 (18)
H2A	−0.2440	0.8268	0.9765	0.069*	0.463 (18)
H2B	−0.3847	0.7855	0.9424	0.069*	0.463 (18)
C1'	−0.2038 (13)	0.6528 (9)	0.9316 (8)	0.054 (2)	0.537 (18)
H1'1	−0.2492	0.5982	0.9103	0.065*	0.537 (18)
H1'2	−0.2150	0.6304	1.0017	0.065*	0.537 (18)
C2'	−0.3120 (14)	0.7637 (13)	0.9008 (13)	0.110 (5)	0.537 (18)
H2'1	−0.3615	0.7923	0.9570	0.132*	0.537 (18)
H2'2	−0.4062	0.7567	0.8690	0.132*	0.537 (18)
C3	−0.0883 (3)	0.82076 (19)	0.79882 (16)	0.0448 (5)	
C4	0.0230 (3)	0.69963 (18)	0.83660 (15)	0.0443 (5)	
C5	0.1907 (3)	0.66401 (18)	0.79889 (16)	0.0451 (5)	
C6	0.2440 (3)	0.74792 (19)	0.72391 (16)	0.0474 (5)	
H6A	0.3559	0.7236	0.6991	0.057*	
C7	0.1314 (3)	0.86943 (18)	0.68465 (15)	0.0432 (5)	
C8	−0.0349 (3)	0.90484 (19)	0.72278 (16)	0.0458 (5)	
H8A	−0.1109	0.9854	0.6971	0.055*	
C9	0.4658 (3)	0.5063 (2)	0.8107 (2)	0.0668 (7)	
H9A	0.5240	0.5615	0.8209	0.080*	
H9B	0.4686	0.5137	0.7419	0.080*	
C10	0.5581 (3)	0.3732 (2)	0.86577 (16)	0.0493 (6)	
C11	0.5419 (3)	0.2761 (2)	0.83868 (17)	0.0581 (6)	
H11A	0.4675	0.2931	0.7880	0.070*	
C12	0.6339 (4)	0.1524 (2)	0.8848 (2)	0.0697 (7)	
H12A	0.6209	0.0874	0.8651	0.084*	
C13	0.7425 (4)	0.1264 (2)	0.9585 (2)	0.0699 (8)	
H13A	0.8061	0.0434	0.9885	0.084*	
C14	0.7594 (4)	0.2206 (3)	0.9891 (2)	0.0758 (8)	
H14A	0.8328	0.2021	1.0406	0.091*	
C15	0.6664 (3)	0.3454 (2)	0.9430 (2)	0.0663 (7)	
H15A	0.6772	0.4100	0.9642	0.080*	
C16	0.1943 (3)	0.95607 (19)	0.60541 (15)	0.0453 (5)	
H16A	0.3115	0.9287	0.5893	0.054*	
C17	0.1011 (3)	1.06911 (19)	0.55476 (16)	0.0478 (5)	
H17A	−0.0167	1.0994	0.5686	0.057*	
C18	0.1795 (3)	1.1487 (2)	0.47661 (16)	0.0458 (5)	
C19	0.0915 (3)	1.28298 (19)	0.43388 (15)	0.0431 (5)	
C20	−0.0676 (3)	1.35676 (19)	0.46461 (15)	0.0461 (5)	
C21	−0.1479 (3)	1.4795 (2)	0.41326 (16)	0.0510 (6)	
H21A	−0.2552	1.5247	0.4335	0.061*	
C22	−0.0687 (3)	1.53574 (19)	0.33120 (16)	0.0487 (5)	
C23	0.0905 (3)	1.4708 (2)	0.30072 (16)	0.0528 (6)	
H23A	0.1448	1.5100	0.2470	0.063*	
C24	0.1693 (3)	1.3463 (2)	0.35090 (15)	0.0473 (5)	
C25	−0.0851 (4)	1.7180 (3)	0.19855 (19)	0.0734 (8)	
H25A	0.0211	1.7290	0.2134	0.088*	
H25B	−0.0575	1.6677	0.1531	0.088*	
C26	−0.2259 (6)	1.9197 (3)	0.2108 (3)	0.1194 (14)	
H26A	−0.3122	1.9971	0.1795	0.179*	
H26B	−0.1174	1.9344	0.2126	0.179*	
H26C	−0.2627	1.8876	0.2758	0.179*	
C27	−0.2982 (3)	1.3618 (2)	0.5841 (2)	0.0680 (7)	
H27A	−0.3059	1.3242	0.6533	0.082*	
H27B	−0.3094	1.4495	0.5740	0.082*	
C28	−0.4456 (5)	1.2299 (3)	0.5652 (3)	0.0982 (11)	
H28A	−0.5417	1.2315	0.5308	0.147*	
H28B	−0.4649	1.2023	0.6337	0.147*	
H28C	−0.3402	1.1729	0.5478	0.147*	

### Atomic displacement parameters (Å^2^)

**Table:**

	*U*^11^	*U*^22^	*U*^33^	*U*^12^	*U*^13^	*U*^23^
O1	0.0523 (10)	0.0408 (9)	0.0614 (10)	−0.0101 (7)	0.0183 (7)	0.0037 (7)
O2	0.0496 (9)	0.0402 (9)	0.0694 (11)	−0.0047 (7)	0.0203 (8)	−0.0036 (7)
O3	0.0429 (9)	0.0350 (8)	0.0643 (10)	−0.0012 (6)	0.0149 (7)	0.0053 (7)
O4	0.0442 (9)	0.0503 (10)	0.0700 (11)	−0.0021 (7)	0.0110 (8)	0.0056 (8)
O5	0.0641 (11)	0.0434 (9)	0.0637 (11)	−0.0075 (8)	0.0032 (8)	0.0065 (8)
O6	0.0993 (15)	0.0432 (10)	0.0642 (11)	−0.0155 (9)	−0.0202 (10)	0.0038 (8)
O7	0.0589 (10)	0.0522 (10)	0.0613 (10)	−0.0085 (8)	0.0240 (8)	0.0028 (8)
O8	0.0620 (12)	0.0685 (13)	0.1118 (17)	−0.0101 (10)	0.0126 (11)	−0.0240 (12)
O9	0.0468 (10)	0.0562 (10)	0.0720 (11)	−0.0076 (8)	0.0192 (8)	0.0028 (8)
C1	0.064 (7)	0.089 (8)	0.065 (7)	−0.001 (5)	0.024 (5)	0.003 (5)
C2	0.043 (5)	0.047 (4)	0.061 (5)	−0.006 (3)	0.025 (3)	−0.003 (3)
C1'	0.042 (3)	0.043 (3)	0.060 (5)	−0.013 (2)	0.016 (4)	0.007 (3)
C2'	0.053 (5)	0.092 (7)	0.125 (10)	−0.012 (4)	0.027 (5)	0.036 (7)
C3	0.0383 (11)	0.0347 (11)	0.0536 (13)	−0.0068 (9)	0.0085 (9)	−0.0094 (9)
C4	0.0463 (12)	0.0347 (11)	0.0464 (12)	−0.0138 (9)	0.0078 (9)	−0.0051 (9)
C5	0.0432 (12)	0.0310 (10)	0.0515 (13)	−0.0066 (9)	0.0063 (9)	−0.0054 (9)
C6	0.0399 (11)	0.0366 (11)	0.0544 (13)	−0.0068 (9)	0.0099 (9)	−0.0056 (9)
C7	0.0433 (12)	0.0352 (11)	0.0464 (12)	−0.0114 (9)	0.0033 (9)	−0.0067 (9)
C8	0.0437 (12)	0.0295 (10)	0.0533 (13)	−0.0056 (9)	0.0052 (9)	−0.0041 (9)
C9	0.0472 (14)	0.0437 (13)	0.0808 (18)	−0.0031 (11)	0.0211 (12)	0.0047 (12)
C10	0.0377 (11)	0.0393 (12)	0.0558 (14)	−0.0047 (9)	0.0124 (10)	−0.0038 (10)
C11	0.0586 (15)	0.0560 (15)	0.0532 (14)	−0.0139 (12)	0.0013 (11)	−0.0113 (11)
C12	0.083 (2)	0.0442 (14)	0.0769 (18)	−0.0134 (13)	0.0072 (15)	−0.0205 (13)
C13	0.0599 (16)	0.0448 (14)	0.0790 (19)	0.0017 (12)	0.0042 (14)	−0.0014 (13)
C14	0.0631 (18)	0.077 (2)	0.0727 (18)	−0.0190 (15)	−0.0193 (14)	0.0036 (15)
C15	0.0725 (18)	0.0574 (16)	0.0744 (18)	−0.0275 (13)	0.0030 (14)	−0.0194 (13)
C16	0.0419 (12)	0.0372 (11)	0.0493 (13)	−0.0102 (9)	0.0057 (9)	−0.0056 (9)
C17	0.0381 (11)	0.0385 (11)	0.0556 (13)	−0.0096 (9)	0.0052 (9)	−0.0011 (10)
C18	0.0373 (11)	0.0435 (12)	0.0479 (12)	−0.0107 (9)	0.0007 (9)	−0.0031 (9)
C19	0.0356 (11)	0.0407 (11)	0.0451 (12)	−0.0105 (9)	0.0012 (9)	−0.0030 (9)
C20	0.0430 (12)	0.0420 (12)	0.0461 (12)	−0.0138 (9)	0.0065 (9)	−0.0037 (9)
C21	0.0439 (12)	0.0424 (12)	0.0542 (14)	−0.0050 (10)	0.0072 (10)	−0.0074 (10)
C22	0.0504 (13)	0.0365 (11)	0.0495 (13)	−0.0112 (10)	−0.0042 (10)	0.0003 (9)
C23	0.0500 (13)	0.0469 (13)	0.0483 (13)	−0.0153 (11)	0.0071 (10)	0.0035 (10)
C24	0.0389 (12)	0.0475 (13)	0.0492 (13)	−0.0153 (10)	0.0058 (9)	−0.0047 (10)
C25	0.0837 (19)	0.0560 (16)	0.0585 (16)	−0.0155 (14)	−0.0043 (13)	0.0096 (12)
C26	0.211 (5)	0.061 (2)	0.090 (2)	−0.051 (2)	−0.006 (3)	−0.0159 (18)
C27	0.0657 (18)	0.0594 (16)	0.0700 (17)	−0.0178 (13)	0.0291 (14)	−0.0172 (13)
C28	0.090 (2)	0.082 (2)	0.130 (3)	−0.0292 (18)	0.019 (2)	−0.044 (2)

### Geometric parameters (Å, °)

**Table:**

O1—C4	1.369 (2)	C9—C10	1.497 (3)
O1—C1'	1.413 (10)	C9—H9A	0.9700
O1—C1	1.425 (14)	C9—H9B	0.9700
O2—C3	1.366 (2)	C10—C11	1.358 (3)
O2—C2'	1.452 (11)	C10—C15	1.384 (3)
O2—C2	1.458 (10)	C11—C12	1.385 (3)
O3—C5	1.377 (2)	C11—H11A	0.9300
O3—C9	1.416 (3)	C12—C13	1.350 (4)
O4—C18	1.245 (2)	C12—H12A	0.9300
O5—C22	1.352 (3)	C13—C14	1.357 (4)
O5—C25	1.437 (3)	C13—H13A	0.9300
O6—C25	1.362 (3)	C14—C15	1.396 (4)
O6—C26	1.400 (4)	C14—H14A	0.9300
O7—C20	1.370 (2)	C15—H15A	0.9300
O7—C27	1.415 (3)	C16—C17	1.319 (3)
O8—C27	1.341 (3)	C16—H16A	0.9300
O8—C28	1.455 (4)	C17—C18	1.477 (3)
O9—C24	1.340 (2)	C17—H17A	0.9300
O9—H9C	0.8200	C18—C19	1.464 (3)
C1—C2	1.264 (19)	C19—C20	1.413 (3)
C1—H1A	0.9700	C19—C24	1.422 (3)
C1—H1B	0.9700	C20—C21	1.375 (3)
C2—H2A	0.9700	C21—C22	1.387 (3)
C2—H2B	0.9700	C21—H21A	0.9300
C1'—C2'	1.282 (15)	C22—C23	1.373 (3)
C1'—H1'1	0.9700	C23—C24	1.384 (3)
C1'—H1'2	0.9700	C23—H23A	0.9300
C2'—H2'1	0.9700	C25—H25A	0.9700
C2'—H2'2	0.9700	C25—H25B	0.9700
C3—C8	1.384 (3)	C26—H26A	0.9600
C3—C4	1.388 (3)	C26—H26B	0.9600
C4—C5	1.395 (3)	C26—H26C	0.9600
C5—C6	1.374 (3)	C27—H27A	0.9700
C6—C7	1.398 (3)	C27—H27B	0.9700
C6—H6A	0.9300	C28—H28A	0.9600
C7—C8	1.388 (3)	C28—H28B	0.9600
C7—C16	1.465 (3)	C28—H28C	0.9600
C8—H8A	0.9300		
			
C4—O1—C1'	112.0 (4)	C12—C11—H11A	119.3
C4—O1—C1	113.1 (6)	C13—C12—C11	119.9 (2)
C1'—O1—C1	26.9 (7)	C13—C12—H12A	120.0
C3—O2—C2'	114.7 (5)	C11—C12—H12A	120.0
C3—O2—C2	110.1 (5)	C12—C13—C14	120.4 (3)
C2'—O2—C2	30.5 (7)	C12—C13—H13A	119.8
C5—O3—C9	116.05 (16)	C14—C13—H13A	119.8
C22—O5—C25	117.68 (19)	C13—C14—C15	119.8 (3)
C25—O6—C26	113.9 (3)	C13—C14—H14A	120.1
C20—O7—C27	120.56 (18)	C15—C14—H14A	120.1
C27—O8—C28	113.5 (2)	C10—C15—C14	120.2 (2)
C24—O9—H9C	109.5	C10—C15—H15A	119.9
C2—C1—O1	122.3 (10)	C14—C15—H15A	119.9
C2—C1—H1A	106.8	C17—C16—C7	126.4 (2)
O1—C1—H1A	106.8	C17—C16—H16A	116.8
C2—C1—H1B	106.8	C7—C16—H16A	116.8
O1—C1—H1B	106.8	C16—C17—C18	121.5 (2)
H1A—C1—H1B	106.6	C16—C17—H17A	119.2
C1—C2—O2	123.6 (9)	C18—C17—H17A	119.2
C1—C2—H2A	106.4	O4—C18—C19	119.96 (18)
O2—C2—H2A	106.4	O4—C18—C17	117.31 (19)
C1—C2—H2B	106.4	C19—C18—C17	122.70 (18)
O2—C2—H2B	106.4	C20—C19—C24	115.78 (19)
H2A—C2—H2B	106.5	C20—C19—C18	126.62 (18)
C2'—C1'—O1	126.8 (8)	C24—C19—C18	117.56 (18)
C2'—C1'—H1'1	105.6	O7—C20—C21	122.18 (19)
O1—C1'—H1'1	105.6	O7—C20—C19	115.90 (18)
C2'—C1'—H1'2	105.6	C21—C20—C19	121.86 (19)
O1—C1'—H1'2	105.6	C20—C21—C22	119.9 (2)
H1'1—C1'—H1'2	106.1	C20—C21—H21A	120.0
C1'—C2'—O2	119.3 (8)	C22—C21—H21A	120.0
C1'—C2'—H2'1	107.5	O5—C22—C23	123.9 (2)
O2—C2'—H2'1	107.5	O5—C22—C21	115.3 (2)
C1'—C2'—H2'2	107.5	C23—C22—C21	120.8 (2)
O2—C2'—H2'2	107.5	C22—C23—C24	119.27 (19)
H2'1—C2'—H2'2	107.0	C22—C23—H23A	120.4
O2—C3—C8	117.66 (18)	C24—C23—H23A	120.4
O2—C3—C4	122.02 (18)	O9—C24—C23	116.51 (18)
C8—C3—C4	120.32 (18)	O9—C24—C19	121.29 (19)
O1—C4—C3	122.35 (18)	C23—C24—C19	122.19 (19)
O1—C4—C5	118.11 (18)	O6—C25—O5	107.9 (2)
C3—C4—C5	119.49 (18)	O6—C25—H25A	110.1
C6—C5—O3	125.25 (18)	O5—C25—H25A	110.1
C6—C5—C4	120.08 (19)	O6—C25—H25B	110.1
O3—C5—C4	114.67 (17)	O5—C25—H25B	110.1
C5—C6—C7	120.63 (19)	H25A—C25—H25B	108.4
C5—C6—H6A	119.7	O6—C26—H26A	109.5
C7—C6—H6A	119.7	O6—C26—H26B	109.5
C8—C7—C6	119.15 (18)	H26A—C26—H26B	109.5
C8—C7—C16	122.12 (19)	O6—C26—H26C	109.5
C6—C7—C16	118.73 (19)	H26A—C26—H26C	109.5
C3—C8—C7	120.32 (19)	H26B—C26—H26C	109.5
C3—C8—H8A	119.8	O8—C27—O7	113.2 (2)
C7—C8—H8A	119.8	O8—C27—H27A	108.9
O3—C9—C10	109.76 (17)	O7—C27—H27A	108.9
O3—C9—H9A	109.7	O8—C27—H27B	108.9
C10—C9—H9A	109.7	O7—C27—H27B	108.9
O3—C9—H9B	109.7	H27A—C27—H27B	107.8
C10—C9—H9B	109.7	O8—C28—H28A	109.5
H9A—C9—H9B	108.2	O8—C28—H28B	109.5
C11—C10—C15	118.2 (2)	H28A—C28—H28B	109.5
C11—C10—C9	120.4 (2)	O8—C28—H28C	109.5
C15—C10—C9	121.4 (2)	H28A—C28—H28C	109.5
C10—C11—C12	121.4 (3)	H28B—C28—H28C	109.5
C10—C11—H11A	119.3		

### Hydrogen-bond geometry (Å, °)

**Table:**

*D*—H···*A*	*D*—H	H···*A*	*D*···*A*	*D*—H···*A*
O9—H9C···O4	0.82	1.71	2.448 (2)	149
C6—H6A···O9^i^	0.93	2.57	3.426 (3)	154
C21—H21A···O8^ii^	0.93	2.53	3.453 (3)	169

Symmetry codes: (i) −*x*+1, −*y*+2, −*z*+1; (ii) −*x*−1, −*y*+3, −*z*+1.
